# Values of welfare technologies: a qualitative study of how employees in Swedish care for older adults understand and justify the use of new technology

**DOI:** 10.1186/s12913-024-12053-1

**Published:** 2024-12-06

**Authors:** Doris Lydahl, Anna Davidsson

**Affiliations:** 1 Department of Philosophy, Linguistics and Theory of Science, Faculty of Humanities, University of Gothenburg, Gothenburg, Sweden; 2 Department of Sociology and Work Science, Faculty of Social Sciences, University of Gothenburg, Gothenburg, Sweden

**Keywords:** Welfare technology, Care for older people, Values, Situated judgements

## Abstract

**Background:**

In Swedish care for older adults, the use of welfare technology − for example, medicine-dispensing robots and GPS alarms − stimulates ongoing evaluations and negotiations. National policy documents, as well as previous research, indicate that these technologies are expected to improve the quality and efficiency of care but also involve potential or experienced challenges in providing high-quality care and the conditions for care workers − often portrayed as a conflict between political and caring organizational values. More research is thus needed on how the use of welfare technology is justified at central and municipal government levels as well as within care-providing organizations. This article aims to identify how the introduction and use of welfare technology is justified by employees and in policy documents pertaining to welfare technology in Swedish care for older people.

**Methods:**

Qualitative interview data involving 37 individuals were collected in municipalities in southern Sweden. The interviewees were managers, administrative staff, and licensed practical nurses working in home care services or special housing for older adults, offering care 24/7. Policy documents and agreements of significance for Swedish welfare technology policy were analysed. The analysis focused on values promoted as significant for the provision of care and related to the use of welfare technology. Justifications and values were analysed as belonging to different ‘worlds of worth’, as either shared or conflicting, and contextually situated.

**Results:**

The documents reflect values regarding, for instance, *efficiency*,* independence*,* good working conditions*, *optimised use of human resources*, and *time to tend to relationships*. The employees justified the use of welfare technologies with reference to *optimisation and efficiency*,* activation and participation*,* dignity and freedom*,* social relationships*, and *good working conditions.* These values represent three different worlds of worth − the industrial world, the civic world, and the domestic world − and are situated in employees’ everyday work practices.

**Conclusions:**

Values presented by employees and in policy documents are shared rather than conflicting. However, employees emphasise values associated with the domestic world, downplaying industrial values. We argue that politicians and civil servants should consider the situated judgements made by employees and develop more bottom-up strategies. This requires an acknowledgement of the existing values hierarchy, where values of optimisation and efficiency are held in high esteem but to accommodate higher values rather than as end goals.

**Supplementary Information:**

The online version contains supplementary material available at 10.1186/s12913-024-12053-1.

## Background

In the Scandinavian countries, digital technologies are introduced and used in health and social care using the notion of ‘welfare technology’ (WT), defined as digital technologies intended to contribute to the welfare of the population by increasing safety, activity, participation and independence for people who have, or risk developing, a disability [1]. While the international literature discusses similar technologies in terms of assistive technology [1], telecare [2], and telehealth [3], the use of the welfare technology concept is intrinsically linked to the notion of welfare, which is shaped by the Nordic care regime and the evolving ideals of the Nordic welfare state. A cornerstone of the Nordic care regime is universality, characterised by equal inclusion through legislated rights, public (tax-based) funding, public provision of services, and the comprehensive, needs-based delivery of quality care services [4]. Equal access to efficient care services has been upheld as a universal social right and regarded as a public good among other tax-funded services as well. This commitment to universality has spawned an extensive system of public care services, which are only supplemented by family and other close relationships [5].

In Sweden, this care regime − particularly in the provision of healthcare and care for older adults − faces significant challenges, primarily due to limited financial resources and the difficulty of staffing with enough competent personnel to care for an ageing population. Facing these challenges, WT has been described ‘as the prerequisite for meeting the demands of future Swedish homecare and healthcare’ [6], with extensive public funds being allocated to stimulate digital development in care for older adults during the last decade.

In 2016, the Swedish Government and the Swedish Association of Local Authorities and Regions (SALAR) agreed on a vision for the development of WT and eHealth. According to this agreement, by 2025 Sweden should be world-leading in benefiting from opportunities for digitalisation [7]. Despite this high-toned ambition, Sweden’s approach to WT in government is less comprehensive compared to the other Nordic countries [8]: there is a lack of systematic coordination, a broad introduction of WT is happening only slowly, and a relatively large percentage of the WTs available in Swedish municipalities today are still part of pilot projects [9]. In practice, it is up to the municipalities to decide what technologies to use and each municipality must develop its own governing documents and action plans, Thus far, however, only approximately 60% have produced such policies [9]. This means that the use of WT in Swedish care for older adults is still a topic of ongoing evaluation and negotiation.

Against this background, we find it important to improve the understanding of how the implementation and use of WT is justified, not only at the central government level but also within the care-providing organisations at the municipal level. Previous research focusing on the users of WT, i.e. older adults and persons living with disabilities, indicates that the technologies are expected to increase the user’s autonomy, independence, safety and well-being [10–17], while there are also concerns about negative effects in terms of less human contact [15, 18]. Focusing instead on the health and social care workers, studies show that WT may facilitate the execution of tasks and assist in the management of staff shortages, thereby positively affecting working conditions [10]. However, within this group there are also concerns about WT negatively influencing work practices with more stress and fewer opportunities for social contact [19]. In summary, there is both optimism and pessimism about what the use of WT might lead to, reflecting different values and ambitions, which may be in conflict with each other (cf. [19–22]).

A basic assumption for this study is the following. If and how technologies are introduced and used in an organisation, or for specific tasks, does not depend on the technology itself. Instead, it depends on many contextual factors, not least the competence and values of those who are supposed to use it. To explore how WT can enhance organizational performance, it is crucial to understand how personnel in Sweden’s health and social care sector perceive WT in relation to the core values of their work practices. However, there are few studies on this topic, making this study a valuable contribution to our understanding.

One way to capture how WT is motivated and perceived in relation to the goals and ambitions of the welfare state in general, and to the provision of care for older adults in particular, is to draw upon the theoretical framework of Boltanski and Thevenot [7]. While this framework will be introduced in detail later, a short introduction includes the key concepts of *justification*, *values*, *worlds of worth*, and *situated judgements*. To begin with, justifications communicate values. To justify something is to give a response about a situation concerning what one finds good, proper, and desirable for the community; in this case, that which is proper and desirable for the organisation of care for older adults. According to Boltanski and Thévenot [9], different values can be understood as related to different worlds of worth, distinguished by what higher principles are used for justifications in various situations. For instance, what Boltanski and Thevenot denominate as ‘the market world’ is based on a higher principle of rivalry and competition, while ‘the civic world’ is guided by principles of solidarity and the collective. Higher principles are manifested in the confrontation between different worlds of worth, when the preferred action needs to be justified, which is done in reference to that which is valued. What principles are supported, and which justifications are made are thus a result of situated judgements, where evaluations depend on the situation (i.e. ‘the world’) in which the justification is made.

The specific aim of this study is to explore how personnel in public organizations providing care for older adults at the municipal level in Sweden justify the use of WT in comparison to the justifications made in policy documents of significance for Swedish WT policy. A general aim is to shed light on potential value conflicts surrounding the use of WT, as well as situated judgements affecting the evaluation of WT, which is important for understanding how the introduction and use of WT can be improved.

The study is based on a document analysis of central policy documents, and qualitative interviews with managers, administrators, and licensed practical nurses in three municipalities. In the analysis, we respond to the following questions:

1. In policy documents with significance for Swedish WT policy, which values are communicated to justify the use of WT and which ‘worlds of worth’ do these values correspond to?
2. Among health and social care workers, which values are communicated to justify the use of WT in the work practices of their organisations and which worlds of worth do these values correspond to?
3. Do justifications expressed in policy documents and among health and social care workers reflect shared or conflicting worlds of worth?

The following text begins with an overview of the Swedish context and previous research. It then presents the theoretical framework of the study, followed by a description of the study’s methodological setting and design. The Results section addresses the first two research questions, while the third question is discussed in the Discussion section. Finally, the results are summarized in the Conclusion.

### Context

In Sweden, health and social care for older adults aims to help both older adults and people with disabilities to live independently. Home care services support this by enabling older adults to live in their own homes for as long as possible. These services typically include domestic help, personal care, medical tasks like medication management, and emotional support [23].

When living at home is no longer feasible, assessed individuals can move into special housing with 24/7 care. This special housing offers comprehensive care including meals, medical attention, and communal living spaces. Individuals living in special housing have an individual lease for their own apartment with a bathroom and kitchenette, and they bring their own furniture and personal belongings. Care needs assessments and decisions are based on their individual needs and in accordance with the Swedish Social Services Act (2001:453).

Care for older adults has changed dramatically in recent decades due to an ageing population, ageing-in-place policies, and pressure to contain costs [24]. Moreover, the number of hospital beds has been reduced, meaning that ‘older people more often leave hospital with remaining care needs, which in turn has increased the demands for municipal eldercare service’ [23]. In addition, the number of older people living in special housing facilities with round-the-clock care has decreased rapidly [25].

In Sweden, the staff performing homecare services or working in special housing are ‘registered nurses’ or ‘assistant nurses’, with the major group being assistant nurses, who are either ‘licensed practical nurses’ or ‘nurse aides’. Registered nurses have a three-year university education in nursing, licensed practical nurses have three years of upper secondary education focused on care and social services, and nurse aides have limited or no education in caring. Assistant nurses are mainly responsible for providing basic care and domestic work tasks, while registered nurses are responsible for medical care.

The management of WT at the municipal level involves several decision-making processes. First, local municipal authorities determine which technologies to procure. Social workers at the social services office then assess and prescribe the appropriate technology for individual users. Additionally, local authorities set the tax rates and budgets, and establish objectives and guidelines related to WT. As a result, there is significant variation in WT usage across different municipalities, which also leads to discrepancies in its adoption and expansion. The types of WT deployed vary not only between municipalities but also among individual users within each municipality. In Sweden, the most commonly used WT is digital social alarms, while other frequently utilised technologies include night-time video surveillance, medicine-dispensing robots, and GPS alarms [9].

### Theory

According to Boltanski and Thévenot’s [26] theories of justification, decisions are made based on different assessments of what, in society, is especially important to attain and preserve. These assessments constitute ‘higher principles’ (also referred to as ‘logics’ or ‘higher values’), which people appeal to when trying to agree upon the ‘best’ or most ‘just’ arrangement of a specific social context. By referring to these higher principles, arguments can be made in a way that, according to Ceci and Purkis [22], ‘enables recourse to a level of generality more defensible than merely personal preference to justify decisions’. It also helps actors engage in the world with confidence [27].

Different higher principles become visible only in relation to each other, particularly when they are considered guiding principles for decision-making. In such confrontations, it is also apparent that certain principles are more common in some contexts than in others. Therefore, the various higher principles are presented as representing different ‘worlds of worth’ (or ‘orders’) in which the value of a person, object or situation is assessed in relation to the higher principles of this world.

Originally, Boltanski and Thévenot [26] presented a typology of six worlds: inspired, domestic, fame, civic, market, and industrial. The various worlds are based on different principles of what is believed to produce a common good. In addition, the worlds are characterized by, for instance, what is renounced (i.e., dystopian) and what is valued [26, 28]. Table 1 presents an overview of the various worlds and some of their characteristics, as exemplified by Boltanski and Thévenot [26].

**Table 1:** The six worlds of worth

World of worth	Higher principle	What is renounced	What is valued
*The Inspired world*	Inspiration	Efficiency, logic, usefulness, rationality, measures, rules, money, hierarchy, and laws	What cannot be controlled or measured, e.g., spontaneity, feelings, and passions
*The Domestic world*	Generation, hierarchy, tradition	Selfishness, bad manners, indiscretion	Relations with others, duty, trust, honesty, and the care owed to those who depend on you
*The Fame world*	Opinion of others	Secrecy, privacy	Recognition, reputation, respect, success
*The Civic world*	Collective, all, general will, solidarity	Individualism, particular and immediate interests	Legality, equal justice, objectivity, freedom, and independence
*The Market world*	Rivalry and competition	Failure, stagnation, rejection	Market position, wealth, success
*The Industrial world*	Efficiency, performance, productivity, capacity	Unproductivity, inactivity, absenteeism, inefficiency, unqualified or ill-adapted	Functionality, reliability, predictability, realistic projects in the future

To understand how these assessments of the common good are translated into practice and coordinated into action, what makes the individual engaged in a disagreement must also be captured. Even though various worlds of worth may coexist, it is through *situated judgements* of these worlds that certain concerns are prioritized and justifications and agreements are made [26, 27].

In this paper, we use this framework to identify the different values that are upheld in policy documents and by care personnel when reasoning about the use of welfare technologies [27]. This is conceived of as their justifications of their preferred use or non-use of WT in their work practice. That is, we analyse what reflects what is valued or renounced in the policy documents and the interview participants’ talk about WT, and what higher principles this represents. By identifying the various worlds of worth that these justifications respond to, we highlight what disagreements surround the use of these technologies. In the analysis, references to these different worlds are thus used to discern which fundamental ideas of ‘the common good’ are represented, and how this may contribute to the presence of value conflicts [27]. 

### Previous research

While there is a lot of international research about the digitalization of health and social care work [1–3], this article specifically builds on and contributes to studies from two research strands: (1) values in health and care work; and (2) the use of WT in health and social care work. This presentation of previous research focuses on these studies, starting with studies that draw on the theoretical framework of Boltanski and Thevenót or that use a similar framework of values, higher principles, and worlds of worth.

As some of these studies illustrate, health and social care workers’ values are not the only values upheld in organizations. Studying a developmental project in municipal care in Sweden, Håkansson [20] points to conflicts and negotiations between what she calls a managerial logic – founded on economic rationales of new public management – and a caring logic that stresses relational aspects and the needs of dependent care recipients. Studying nursing homes for older adults and home care services, Chia [21] contends that care for older people balances efficiency with market-driven logic, limiting the altruistic ‘heart work’ associated with emotional care. Additionally in their study of home care, Ceci and Purkis [22] note a similar dual justification: an industrial logic emphasizing efficiency and a domestic and civic logic valuing personalization, affection and responsiveness. Lehoux et al. [27] examined the interplay between morality and the economy in health technology development, arguing that there is a conflict between economic and organizational worth, which aligns with the higher principle of efficiency, and interactional and clinical worth, which aligns with the higher principle of health and well-being.

Håkansson’s study [20] shows that even though both logics are present in the organization, ‘the managerial logic conditioned the caring logic through the control over resources’. This is congruent with the findings of Björnsdóttir et al. [29], who use a combination of discourse analysis and worlds of worth when analysing the changing practices of government in relation to older people in Iceland. They show that older people are ‘increasingly expected to continue to live in their own homes, caring for themselves with assistance from their relatives, friends and available community services, even though their health and ability may have declined’. The authors concluded that arguments in favour of using a standardized tool for care needs assessment were grounded firstly in the industrial world of worth, prioritizing efficiency and prediction, and secondly in the civic world of emphasizing collective interests and thereby renouncing individuality [20]. Values tied to the industrial order thus seem to trump values associated with other worlds of worth.

Using somewhat different terminology, the studies presented above show similar findings, with conflicts and negotiations between different higher principles influencing how the organizations are run. More specifically, it is the values of the industrial world that appear to stand in opposition to values pertaining to a domestic or civic world of worth.

Research on the second strand – WT in health and social care work – is a growing but limited field. This research highlights the importance of technology functionality and its support for users, i.e., for older adults and persons living with different disabilities. The justifications for WT, which were raised in these studies, reflect a relatively long list of values. It is claimed to increase autonomy, independence and safety [10–15, 19] and may facilitate older people continuing to live at home, to be more active in their own care, to reduce isolation, and to increase well-being [16, 17]. However, there are also concerns raised about the potential loss of core care values with the introduction of digital welfare technologies, including a decrease in human contact [15, 18].

Other studies illustrate health and social care workers’ attitudes and views on the use of WT. For instance, WT facilitates the execution of tasks by reminders and scheduling but also facilitates communication, learning, treatment and remote management, control and information [10]. A more efficient organization of work also helps to manage staff shortages, which positively affects employees’ working conditions. However, there are also studies of health and social care workers that point to the challenges involved in using new technology and of changing established work practices. For instance, Kleiven et al. [30] show how values of effectiveness collide with those of care, and how professionals – used to working independently – are now dependent on technical support. Grosen and Hansen [31] studied the implementation of sensor floors in Danish care for older adults, emphasizing the value conflicts between privacy and security, resulting in increased workload and ethical dilemmas for health and social care workers. Similarly, Lydahl [19] demonstrated how the values of certain forms of togetherness and availability, a stress-free working environment, and functionality may be neglected. Chang et al. [32, 33] focused on care workers’ views on social alarms, revealing the complexity of technology integration and identifying differences in workers’ goals, focus, and coping strategies. Grünenberg et al. [34] attributed resistance to technology becoming ‘monstrous’ and unattractive since its introduction often occurred too quickly, without consideration of sensory-bodily and experience-based aspects of care work.

A common theme in the more critical studies is that the different actors involved in the design, implementation and use of welfare technologies have different views and perspectives influenced by professional norms and knowledge traditions [35] and that different actors’ values are sometimes in conflict [36]. As argued by Nakrem et al. [37], it is the important to attend to professional judgement when introducing welfare technologies, as the care worker’s perception and judgement of the technology is vital for successful implementation.

## Methods

### Study design

To understand how personnel working in care for older people justify the use of welfare technologies, qualitative semi-structured interviews were performed with managers, administrative staff with responsibilities related to WT, and health and social care workers in three municipalities in southern Sweden. This data was collected as part of a larger project on welfare technologies in health and social care for older adults in Sweden [38]. A document analysis of influential policy documents was undertaken to explore how policy documents and employees’ justifications reflect shared or conflicting worlds of worth.

### Sampling

The interview data was collected from three municipalities in southern Sweden. These municipalities vary in size, demographics, and socioeconomic conditions. This sampling strategy was chosen as it aligned with the aim of the larger project of which this study is part [38].

The first municipality is considerably larger than the others, with nearly 10,000 employees working in municipal health and social care. While this municipality is generally prosperous, it has a slightly higher unemployment rate compared to the national average and a higher percentage of foreign-born residents.

The second municipality is medium-sized, with approximately 4,000 employees in municipal health and social care. It enjoys relatively low unemployment rates and good socioeconomic conditions. Compared to other municipalities of similar size, it has a lower proportion of foreign-born residents.

The third municipality is relatively small, with only 1,400 employees in the municipal health and social care sector. It has low unemployment levels, strong socioeconomic status, and a lower percentage of foreign-born residents than the national average.

All three municipalities use the most common welfare technologies (WTs) such as social alarms, digital camera systems for night-time monitoring, digital locks, and digital systems for medicine administration. However, there is variation in the use of less common WTs, such as technologies for digital purchasing, digital systems for physical activity, and digital medicine dispensing systems. Additionally, the providers of these digital technologies and systems differ across the municipalities.

The municipalities were chosen to represent the specific region of southern Sweden, where the interview study was conducted [38]. While the analysis attended to the differences between the municipalities and their potential effects on the responses, no significant variations in values were found. For the purposes of this study therefore, the interview data is presented as a single, unified dataset.

Interview participants, i.e. managers, administrators and licensed practical nurses, were recruited through managers from each of the municipalities. Managers were asked to recruit both older and younger participants, as well as those with varying levels of experience working with WT. (Further details of the participants are presented below.) Gender, age, ethnicity, education, and work experience were not included as recruitment criteria, as they were not part of the original study design [38]. Although questions about gender, age and work experience were asked during the interviews, the analysis revealed no significant differences in how employees justified the use of WT based on these factors. For this reason, this will not be discussed further in this article.

The analysed policy documents were selected based on their significance. All documents that have had a direct financial and/or political impact on Swedish WT policy, since the concept first was defined in 2013, were included in the dataset. A total of five documents were included: the original 2013 definition of welfare technology, two agreements between the Swedish Government and the Swedish Association of Local Authorities and Regions, one Swedish Government Official Report, and one government bill. These are presented in detail under ‘Results’.

### Interview participants

Participants constitute managers, administrative staff with responsibilities related to WT, and licensed practical nurses. For simplicity, and because the term ‘nurses’ may be wrongly associated with registered nurses, the participants who work as licensed practical nurses are hereafter referred to as ‘care workers’. When talking about the whole sample, the interviewees are referred to as ‘personnel’.

Table 2 presents the characteristics of the interview participants. In total, 37 individuals were interviewed. The composition of the interviewees largely corresponds to the situation in specific home care service units and the special housing units where the interviews were carried out.

In the large and medium-sized municipalities, interviews were conducted with personnel in both home care services and working in special housing for older adults, offering care 24/7, while only personnel in the home care services were interviewed in the small municipality. The fact that fewer interviews were carried out in the small and large municipality had to do with difficulty in recruiting participants due to the ongoing COVID-19 pandemic.

**Table 2:** Characteristics of interview participants

Characteristics	*N* (% ) or Mean
Gender	
* Male*	6 (16.2%)
* Female*	31 (83.8%)
Occupation	
* Care worker*	24 (64.9%)
* Manager*	6 (16.2%)
* Administrative worker*	7 (18.9%)
Place of work	
* Home care services*	17 (45.9%)
* Special housing*	17 (45.9%)
* Municipal wide administration*	3 (8.2%)
Municipality	
* Small municipality*	6 (16.2%)
* Medium-sized municipality*	19 (51.4%)
* Big municipality*	12 (32.4%)
Age	Mean: 47 years
	Range: 19 years – 60 years

### Interviews

Interviews were carried out by DL between September 2020 and June 2021. As the initial interview time sometimes proved insufficient, some participants were interviewed twice, with the final number of interviews being 47 (with 37 interviewees). Most interviews were conducted individually, except for four individuals who were interviewed together due to scheduling constraints. Due to the COVID-19 pandemic, all interviews – except for the group interview – were conducted either on Teams or by phone. All interviews conducted on Teams were conducted with the camera on. The interviews lasted between 16 and 71 min. The shortest interviews were with those participants who were interviewed twice. While the form of the interviews differed, the analysis shows that the form did not appear to significantly affect how the interviewees responded.

The interviews included questions about what kind of welfare technologies were used and when and why (for a full interview guide see the supplementary file). Interviewees were also asked about the situations in which WTs were deemed appropriate or inappropriate. In line with Lehoux et al. [27] ‘our assumption is that the claims […] elicited by interviewees draw, to a large extent, on socially shared normative repertoire’. Consequently, the interviews are seen as a forum for justifications, where interviewees need to explain and justify the use of these technologies.

### Analysis

Audio recordings of the interviews were transcribed verbatim, de-identified and imported to Nvivo, a software for qualitative analysis. The policy documents were also analysed using Nvivo. The analysis broadly follows an interpretative thematic analysis approach [39]. Interviews, policy documents and agreements were first coded inductively in a round of data-driven coding [40] where the material was coded for values. Here, a loose definition of values as ‘that which is deemed desirable’ was used. The researchers then discussed this coding and decided to perform a second round of concept-driven coding [40] where the values collected in the first round were thematised according to the ‘higher principles’ and ‘worlds of worth’ presented by Boltanski and Thévenot [26]. Finally, the themes were reviewed [39]. In this reviewing process, some of the justifications coded in the first round were excluded, for instance about using WT to attract younger employees, or about the lock-in effects of procured technology.

In the analysis, gender, age, occupational role, and municipality did not appear significant for how the participants responded, and the results are presented without distinguishing between interviewees based on these factors. All quotes have been translated into English by the authors and edited for readability.

### Ethical considerations

The study was reviewed and approved by the Swedish Ethical Review Authority (Reg. no. 2020 − 01344). All interviewees gave informed written consent to participate in the study. The written consent form explained the risks and benefits of participating in the research and their rights as study participants. Pseudonyms were used throughout the reporting of the findings, and any identifying characteristics were anonymised.

## Results

The results are divided into two sections, corresponding to the two first research questions. The first section presents the justifications found in policy documents and agreements, highlighting the values and corresponding ‘worlds of worth’ they represent. With a similar approach, the second section focuses on the interviewees’ talk of welfare technologies within their work practices.

### National governance and prioritised values

In Sweden, national governance relating to digitalisation in social services and healthcare has existed for almost 20 years. This section presents influential policy documents along with the values – and corresponding worlds of worth – represented in these documents (see Table 3).

**Table 3:** Values attached to the use of welfare technologies as represented in policy documents and agreements

Document	Represented values	World of worth
National Board of Health and Welfare – original definition (2013)	Safety, activity, participation and independence	Civic
Ministry of Health and Social Affairs and SALAR (2016)	Good and equal health and welfare; independence and participation in the life of society	Civic
	(Competition)	(Market)
Ministry of Health and Social Affairs and SALAR (2022)	Efficiency, increased capacity, optimised use of human resources	Industrial
	Activity	Civic
	Person-centeredness time to tend to relationships	Domestic
Swedish Government Official Report (2020)	Optimised use of human resources, flexibility	Industrial
	Sustainable and attractive working conditions	Civic
Govt proposition 2022/23	Optimised use of human resources, flexibility	Industrial
	Quality, security, independence, good working conditions	Civic

All the analysed documents refer to the National Board of Health and Welfare’s definition of WT, first published in 2013. According to this definition, WT is a digital technology which aims to increase safety, activity, participation, and independence for people who have, or are at risk of developing, a disability [41]. These are values related to the civic world of worth as arrangements in the civic world strive to give the collective a ‘body, permanence and presence’ [26]. While drawing on the same definition of WT, the policy documents produced later thus similarly represent values associated with the civic world of worth [42, 43]. For instance, in 2016 the Swedish Government and the Swedish Association of Local Authorities and Regions (SALAR) endorsed a joint vision for eHealth which states that:

*In 2025*,* Sweden will be the best in the world at using the opportunities offered by digitalisation and eHealth to make it easier for people to achieve good and equal health and welfare and to develop and strengthen their resources for increased independence and participation in the life of society.* [7]

This statement begins by advocating the principle of competition – associated with the market world – by proposing that Sweden is to be ‘the best in the world’ at benefiting from digitalisation. This introductory formulation aside, the vision emphasises equality, independence and participation in society, which positions the vision within the civic world of worth: ‘This involves using various types of IT support to activate users’, clients’ and patients’ own resources in order to achieve important values such as improved health and increased participation and self-determination’ [7].

As a next step, financial support to realise this joint vision was introduced. In 2018, the Government decided on a central government grant of SEK 350 million to the municipalities for investments in WT during that year, and in January 2020, the Government entered into a new agreement with SALAR. The *Agreement on care for older people − technology*,* quality*,* and efficiency with the older person in focus* was intended to last for three years and covered further funding of SEK 200 million per year, most of which consisted of a central government grant to the municipalities [44]. In this Agreement, the efficiency and increased capacity of care for older adults, which are values associated with the industrial world of worth, are in focus. However, the Agreement also underscores that WT will make care for older adults more person-centred and free up time for meaningful activities and ‘the meeting between the older person and the care personnel’ [44]. These justifications, valuing time to tend to relationships, connect to the domestic world of worth. In addition, values associated with the civic world are continuously present in the Agreement, not least in the emphasis on meaningful activities.

Also in 2020, a Swedish Government Official Report on WT called *Future Technology in the Service of Care* was published [45]. An important point of departure in the Report is that care for older people faces, and will continue to face, great difficulties in recruiting staff. The report suggests a dual focus: attracting new staff and maximising the potential of current employees through a sustainable and appealing working environment. WT is presented as enabling a flexible organisation of work, granting more time for care tasks, and consequently alleviating stress and minimising repetitive strain injuries. Concerns about safety and working conditions in health and social care belong to the civic world, focusing on providing good working conditions for staff. The idea of freeing up time to let care workers focus on what they do best reflects a value concerning citizens’ well-being, tied to performing tasks confidently. Alternatively, it can be viewed through the lens of the industrial world of worth, aiming for quality results through optimised resource use and a flexible organisation of work.

In 2023, the Swedish Government proposed a bill based on the 2020 Swedish Government Official Report, concerning WT in the care for older people [46]. This text advocates an increased use of WT to manage demographic changes and a shortage of skilled labour. The proposal highlights benefits such as enhanced flexibility in social services interventions, maintaining quality, ensuring that tasks are performed consistently, providing round-the-clock services, and strengthening the individual’s sense of security. Additionally, technology is seen as promoting older persons’ health by enabling independent living and efficient time planning. The Government envisions that technology will reduce employees’ stress by handling routine tasks, emphasising the strategic deployment of care staff where technology cannot replace them [46].

In summary, all the documents highlight values associated with the civic world (i.e. quality, security, independence, and good working conditions), while the justifications for WT presented in the Government Official Report and Government bill proposal, which were produced later, are also strongly linked to the industrial world of worth (i.e. flexibility and an optimised use of resources). This might constitute a potentially growing values conflict between the governing bodies and the actors closer to the practical reality of care of older people.

### Justifications for welfare technologies among health and social care workers

As presented above, the introduction of WT has largely been a result of top-down initiatives and central government funding. The focus in the following is on how individuals ‘on the ground’ respond to these initiatives and how their locally situated judgements are justified by promoting values essential to either the personnel or the older adults [34]. The presentation of the results is structured thematically, reflecting categories of values expressed in the interviews: *optimisation and efficiency*,* activation and participation*,* dignity and freedom*,* social relationships*, and *good working conditions*.

First, a general comment on the results regarding the different occupational groups among the interviewees: administrators, care workers, and unit managers. Given their distinct responsibilities, some variation in how they perceived and justified the use of WT was expected. However, the analysis revealed more similarities than differences in how they discussed WT and the values it promotes. As a result, the presentation of the results does not focus on the interviewees’ occupational groups, except for the administrators, whose primary concerns of optimisation and efficiency will be elaborated in the following section.

### Optimisation and efficiency

In the interviews, the use of WT is recurrently justified in ways that uphold the values of optimisation and efficiency. Sometimes, as in the following extract, the expression of these values is a clear reflection of political rhetoric:

*Interviewer: What have they [the management] said about this*,* what is the reason to start using WT?*

*IP 34: It is probably to make things more efficient*,* I think*,* so that we ourselves can see more clearly*,* so that it is easier for the coordinator as well… I truly don’t know. I think that we are moving into a fully technological society.*

*(IP 34 care worker, home care services)*

In this quote, the care worker discussed the reasons she had heard for the implementation of a new technology for planning home care services. Even though she previously talked about technology being ‘both good and bad’, she repeats the principles of efficiency and simplicity communicated by politicians arguing based on an industrial higher principle. The quote thus demonstrates how care workers learn and represent the values promoted through this principle. By referring to the inevitable development of technology in society, the quote further relates to the industrial world and the principle of realistic future projects.

Furthermore, interviewees view WT as promoting efficient resource utilisation, reflecting situated judgements [27] rooted in their practical experiences rather than top-down initiatives. For example, one challenge for organisations is to optimize schedules for care workers in a way that matches the needs of older people. This was highlighted by administrators, as in the quote below, which illustrates the impossible task of being everywhere at once:

*And for us administrators*,* it will be easier to make the schedules*,* as we don’t have to plan visits at exact times. It is always difficult for us*,* who are responsible for the planning*,* as we have many users who need to have their medication at the same time. Everyone should always have this medicine at eight o’clock*,* for example*,* but it is impossible for everyone to get it at eight o’clock. It will be easier for us if there are more users who have the medication dispenser*,* because then we do not have to schedule the visits for them to get their medication at the exact right time.*

*(IP 2 planning administrator, home care services)*

Using medication dispensers, the difficulty of distributing medicine at the same time to many users is then solved without having to increase the number of staff.

The administrators in particular stressed that WT replaced certain routine tasks, thereby making it possible for them to manage complex organisational conditions and work practices. For instance, the interviewees in one municipality described how the use of medical robots had enabled a reduction in the number of hours they spend on distributing medicine equivalent to four full-time employees in a year. Nevertheless, the interviewees emphasise that these initiatives do not aim to reduce the number of staff; instead, they are meant to address difficulties in finding enough competent staff to provide care to an increasing number of older adults.

Similarly, mobile documentation was introduced in one municipality as a way of meeting the need for increased documentation while also reducing the time that care workers spend waiting to document their visits on the joint computer. As illustrated by the following quote from a system administrator, the possibility of documenting continuously throughout the day is not only about efficiency but also about safety:

*Previously*,* it was clear that a lot was missed in the documentation. In addition*,* it was not a matter of not caring; it was a matter of*,* say*,* someone on the staff going out in the morning and making eight home visits. In addition*,* after four of these visits*,* something happened that was supposed to be documented*,* and then I came in for lunch and I did not have time*,* so I went back to work. In addition*,* I had further visits and even more things happened*,* and then at the end of the day*,* before I went home*,* I had to document everything. Then*,* it is very likely that you forget a lot of things.*

*(IP15, system administrator, central administration)*

The investment in mobile documentation thus compensates for insufficient resources in time and infrastructure to carry out the required tasks, which provides grounds for a situated judgement of this technology as justified.

The integration of various systems (i.e., documentation, signing, key cards, scheduling and planning) in employees’ smartphones was argued to provide increased flexibility for care workers and their execution of tasks. This approach corresponds to the ideas of WT freeing up time and allowing care workers to focus on caring for older people. In addition, the interviewees talk about freeing time resources that can be used for tasks where their competence is more called for, that is, a better allocation of competence.

### Activation and participation

The interviewees often referred to using WT as creating opportunities to improve how they involve, schedule, spend time with, and engage older people in various activities. In this regard, the values traced here relate to activation and participation. These values are interpreted as aligning with the civic world of worth, as the justifications identified in the interviews emphasise providing older people, as a collective, with more opportunities to participate and stay active through WT [7].

Interviewees refer to two types of participation. The first is related to *participation in the care process*, and the other to *participation in society*. Both forms are viewed as something worth striving for in care for older adults in general, but several interviewees also talked about the associated difficulties. The situated judgements found in the interviews show complexities, asking questions about what participation in the care process is and how this participation can be achieved. One of the administrators explains these complexities:

*The first thing that comes to mind is participation. Here I am thinking first and foremost about user participation. This is something that we struggle with all the time*,* or rather strive for*,* I would say. Trying to get users involved. That it is not just about us carrying out an assignment that we have been ordered to do*,* an official decision that must be implemented*,* but it is also about a life and a person who should be allowed to continue living their life. In addition*,* if you can somehow use technology for that*,* I think that is why WT is very much [a good thing].*

*(IP 13 development administrator, central administration)*

The cited administrator emphasises the importance of involving older individuals in care processes, moving beyond mere task execution. This quote underscores the aspiration for technology to enable active participation, allowing older people to maintain their independence and continue living their lives − an aspect further discussed in the upcoming section of the article.

Becoming an active participant in one’s care is a theme found in several interviews. For example, when talking about the introduction of mobile documentation, a manager referred to this kind of participation as a reason why it is good to use WT:

*I think it’s also about the user’s participation*,* that you can write when you’re with the user: ‘Now*,* I notice that you’re worried*,* so now I’m writing it in your documentation’. Because they can request their documentation later. It is the user’s document. That also creates participation.*

*(IP 16 manager of home care service unit)*

By emphasising that the older person owns and can access their documentation, the quoted manager contends that this involvement makes them active participants in the documentation process. This form of participation focuses on preserving individual privacy and serves as a justification for advocating the use of mobile documentation.

The value of activation is thus closely related to the second form of participation. Here, welfare technologies are talked about as important tools for making older persons more active and independent, as the technologies allow them to participate in social activities with other older persons and in *society at large*. This is illustrated in the following quote:

*We should work for the whole picture. The residents should be as involved as possible so that they can also keep up with the times. You can organize activities that they already know*,* but they can also take part in what’s happening now. Today’s older people are quite youthful in a different way.*

*(IP20 development administrator, special housing)*

As examples of activities, the cited administrator mentioned online gymnastics and finding old video clips with music and pictures that the older person remembered. While this could be argued to reflect participation in the care process, it could also be perceived as participation in both senses. While physical activation is indeed part of the care of the older person, the administrator underscores that older people can keep up with the times and take part in what is ‘happening now’. Using WTs, the administrator argues, becomes a way of ensuring that the older person can take part in things ‘we’ take for granted (such as watching video clips on streaming platforms). Implicitly, she thus refers to a sort of ‘universal right’ to participate in a digital society.

### Dignity and freedom

Often overlapping with justifications of activation and participation, interviewees use justifications stressing values of dignity and freedom for older people. These justifications are related to solidarity – associated with a civic world of worth – and compassion, which relates to the domestic world of worth. In the following quote, the use of medication dispensers is presented as making it possible for the user to be independent and active but also increasing her freedom:

*This has worked especially well for one individual who has been able to take the medication herself [by means of the medication dispenser]. Previously*,* we had to open the sachet with medication and put it in egg cups. Now*,* she manages to open it herself and is much more independent in that way. She can leave her home instead of sitting and waiting for home care services to come and help her with her medication three times a day. Now we only visit in the morning*,* which means that she has time to do whatever she likes the rest of the day.*

*(IP 3, care worker, home care services)*

Unlike the examples discussed earlier, activation in this context is not linked to participation. Instead, the focus is on the individual’s freedom to pursue their preferences: ‘to do whatever she likes’. This justification aligns with a liberal tradition that values individual freedom as crucial for a fulfilling and meaningful life, where freedom constitutes a basic civil right [26].

Commonly, freedom is described as protecting the individual’s ‘dignity’, illustrated in the following excerpt:

*The first thing that comes up is a future where we have… a dignified way of helping people who live here. By dignified*,* I mean that you do not have to be woken up by night staff*,* but we have solved it with security technology and people should have the right to go out and walk around whenever they want and have the feeling that they want to go home. You should be able to go straight out through a door*,* but we should be able to have a system where you know where the person is going if they cannot find their way back. Today*,* with some people*,* it is very difficult to keep it dignified enough to… you have to make restrictions*,* and we never do that using a door*,* but we… we have to ask where they are going*,* we have to follow them. And I think that the future can give us better solutions to that. I look forward to that. That is the first thing I think of with WT*,* there’s so much more*,* but in my organization*,* that is what we’re looking forward to.*

*(IP 21, manager of special housing unit)*

According to this manager, dignity encompasses the right to remain undisturbed at night and the freedom to walk outside at one’s convenience − highlighting both privacy and personal freedom. The concept of freedom is theorised to be closely linked to dignity, viewing freedom as a prerequisite for dignity [26]. In the civic world of worth, human dignity is considered a civil right [47]. The manager acknowledges the current challenges in ensuring these rights for safety reasons and expresses optimism about future technological solutions that could enhance the individual’s freedom while maintaining their safety.

In special housing with 24/7 care for older persons with dementia, the use of GPS alarms provides an example of technology that allows them to let the doors remain open to the older person. In doing so, they are complying with legal restrictions (which prohibit them from locking doors), and increasing personal freedom while also monitoring older persons. Similarly, nightly video monitoring enables care workers to avoid frequent or unwanted visits. The use of these technologies thus still involves a high level of monitoring. In contrast to institutional housing with locked doors, WT may grant the user an increased sense of personal freedom. As older persons are simultaneously monitored, this freedom can be understood as illusory. Care workers described it as a compromise between respecting dignity and fulfilling work tasks by maintaining control.

### Social relationships

Some of the interviewees justify the use of WTs by arguing that it creates more time for social interactions and human connection. In addition, many care workers see the technology as a facilitator of relationship building. For example, tablet computers in home care services can enhance interaction with older individuals, enabling them to participate in activities such as video calls with family members. The underlying values revolve around fostering connections with others and providing care for those dependent on you, which are related to the domestic world.

At times, the care workers made situated judgements in favour of adopting WT, as it enabled them to engage in significant relationship-building activities that would have otherwise been inaccessible to the older individuals they were assisting. In the following example, the care worker highlights the use of technology to enable a sick older woman to virtually attend her son’s funeral through the care worker’s work phone:

*There was a woman who was very ill*,* her son was going to be buried*,* and she could not attend the funeral in person because she was dying herself. However*,* she was still allowed to attend the funeral because we had video calls with her relatives. During the placing of the urn and all that*,* she was with them*,* but not quite*,* and I sat and held her*,* and the tears flowed. You know*,* it was kind of cool to be able to offer that.*

*(IP 36, care worker, home care services)*

The above quote illustrates the justification for technology use as twofold: first, it allows the older woman to maintain relationships and to exercise her societal right to participate in a funeral despite being unwell. Second, the video call strengthens the bond between the older woman, her family, and the care worker, bridging the domestic and civic spheres.

Another situated judgement for the use of WT was the recognition of its capacity to stimulate users while also serving as a valuable catalyst for conversation, fostering social interactions between care workers and users, as well as among the users themselves. Several care workers in special housing mentioned the use of digital map tools as an example of this phenomenon, which is illustrated in the following quote from one of them:

*We have been travelling − I was about to say − in our imagination*,* in different countries. We have looked at the world with Google Earth. They [the older persons] had to name a country they wanted to visit*,* and then they had to look*,* and I had to go in and read about different things they wanted to know. […] It was also the same week as the cultural week here in the city*,* so I talked a bit about artists too. So it was like: where can we find this Michelangelo painting*,* in which country? So I looked up the country. They thought it was fantastic*.

***(****IP 7 care worker*,* special housing)*

In the example above, the specific technology is described as bringing joy, community, information and a sense of connectedness to the outer world. Therefore, this is more loosely connected to the domestic world, as it is not about building meaningful and deep relationships, but about having a good time and feeling connectedness. Nevertheless, the analysis shows that the care worker’s perspective asserts that the technology helped in gaining a deeper understanding of older individuals while simultaneously offering them an opportunity for socialisation and shared enjoyment.

Several arguments for not wanting to use WT and where the technologies are judged to be not valuable were also provided in the interviews. These are situated judgements based on technology − in some situations − being perceived as detrimental to the relationship with the older person:

*It depends on whether you are talking about WT that replaces people. We must be very careful about that. But… as we are talking about safety during the night*,* we are extremely careful to look at the individual*,* where − for some people − it is fantastic that there is a camera that turns on when they move in a way they do not usually move. For others*,* it is very important that a person comes and pats them on the cheek.*

*(IP 21 manager of special housing unit)*

In this quote, the manager highlights the importance of seeing the individual’s needs. For some people, a camera for night-time monitoring may be good, but for others, a care worker checking in on them is preferred. The type of judgement made is thus dependent on the individual context. The same type of reasoning can be found in the quote below, where the care worker discusses how some forms of WT can be good, while other technology could contribute to a sense of insecurity and lack of humanity and relationship:

*IP 22: Everything that has come now*,* such as the social alarms that they can press a button on and a person comes […] It’s a great sense of security for them*,* absolutely. The only thing you think about is AI in the future*,* what it will be like. It’s a bit… what they will be able to do*,* it’s another matter if it goes that far. […] There will be no closeness. It will probably be difficult for AI to take over the feeling that there is a person you like and feel safe with.*

*(IP 22, care worker, special housing)*

The cited care worker’s arguments connect to values related to the domestic world of worth. Knowing and feeling that there is a person who you feel safe with is judged to be important both for the provision of care and the older adults themselves, while technology − specifically artificial intelligence − is not expected to provide that kind of feeling.

### Good working conditions

WT use was explicitly justified by the collective benefit to personnel. Interviewees frequently highlighted how WTs eased certain tasks, reducing mental stress. Such judgements span both the industrial and civic worlds of worth, emphasising efficiency as well as the well-being of the staff [26]. Using digital signing for medication, for instance, is described as making this work task ‘nicer’ by allowing a better overview of the medicines and an indication of whether something is wrong. The possibility of ‘freeing up time’ for social interaction is also advocated from the employee perspective, as human relationships are viewed as a rewarding aspect of the job. In the following excerpt, a care worker expresses hope that WT will provide additional time for meaningful interactions with older individuals:

*Not everything can be done with technology either. In my profession*,* the human being must be priority one*,* which. It is also that you wish you had slightly more time. A few minutes. That’s what it is. Then*,* hopefully*,* technology can help us get these minutes if politicians do not want to give it to us. That is my hope.*

*(IP 35 care worker, home care services)*

A perceived value conflict emerges regarding WT’s impact on relationship-building and improving working conditions. A care worker highlights that ‘not everything can be done with technology’, suggesting that WT should not overly replace human workers, as some tasks require a human touch. Despite this, she justifies WT by hoping it will allow her more time with the older person, linking it to values of improved working conditions and social connection. She further argues that politicians, focused on balancing the budget, are unwilling to provide this time, implying they prioritise different values. The conflict, as perceived by the interviewee, lies between the industrial world − where WT is justified by financial efficiency and replacing workers − and the civic and domestic worlds or worth, which prioritise good working conditions and human relationships.

However, the value of good working conditions was also raised to justify a critical stance to WT. This involved talk of technology-related stress (e.g. by being logged out of systems) and increased work stress. This is illustrated in the following quote by a care worker talking about how the use of social alarms increases stress for staff and older persons alike:

*This is a working environment problem because it is stressful and difficult. You’re in someone’s apartment who is maybe very anxious and who is having a bad morning and the only thing that happens is beeps and pings*,* and older people become even more stressed and feel even worse*,* but there’s nothing we can do about it.*

*(IP 23 care worker, special housing)*

As presented by this care worker, the use of WT has led to intensely stressful situations in which the older person feels anxious, and the care worker’s working environment is worsened. A good working environment is a principle related to the civic world of worth, as it has to do with labour rights. However, this principle, as in the previous example, also overlaps with principles relating to the domestic world of worth, as the care worker finds it difficult to get through to the anxious older person with all the constant beeps and pings, thereby finding it difficult to care in the way she would like to.

## Discussion

The presentation of results has shown how the introduction and use of WTs in Swedish care for older people is justified in policy documents and by health and social care workers, and the worlds of worth these justifications represent. This Discussion section primarily attends to the third research question regarding whether the justifications expressed in policy documents and interviews reflect shared or conflicting worlds of worth. In relation to previous research, it also shows how justifications are situated in occupational practice and within a hierarchy of worth. In addition, it discusses the implications the findings of this study may have for the introduction of WT in care for older people.

### Shared or conflicting worlds of worth?

Previous research has shown that healthcare and social care constitutes an arena where values associated with different worlds of worth clash. For instance, how care as a value is often emphasised as being in competition with the demands of the industrial world for increased efficiency [29] and that this limits the ‘heart work’ of eldercare [21]. Similarly, Håkansson argues that a managerial logic conditions a caring logic in the organisation [20].

In contrast to previous research [30, 31], this study’s analyses of policy documents and interviews do not provide many examples of conflicting values. Justifications of WT, as presented by the interviewees, represent values categorized into optimisation and efficiency, activation and participation, dignity and freedom, social relationships, and good working conditions. These values are also represented in the analysed policy documents, and correspond to the higher principles of the industrial, civic, and domestic worlds of worth. In conclusion, care personnel and producers of policy documents appear to be acting in shared worlds of worth, with similar judgements on what higher principles should be guiding the organisation of care for older people.

While this conclusion relates to shared values between policy documents and personnel, the interview material provides examples which indicate that personnel may still perceive that there are value conflicts in the organisation of care of older people. For instance, one of the care workers stresses that technology cannot substitute for human workers, but still helps by allowing the re-allocation of time spent on different tasks and freeing up extra time that ‘politicians do not want to give us’. Thereby, this interviewee seems to imply that there is a conflict between the industrial world (represented by politicians), where WT is justified in relation to financial and budgetary values by providing opportunities to replace human workers instead of paying for more staff; and the civic and domestic worlds of worth (represented by the care workers) prioritising good working conditions and social relationships. It is possible that a different study − one that looked at the politicians at the municipal level instead of the governing policy documents − would find that this perceived conflict is real.

In summary, although conflicts might exist between the higher principles associated with the industrial, civic and domestic worlds of worth [26], care personnel and policy documents share these principles and thus seem to be equally split between these worlds.

### Situated judgements

The findings of this study concerning upheld values are congruent with much of the previous research on the use of WTs in health and social care work. As in previous research, the results show that participation, autonomy and independence are important values in the justification of WT [11−16]. Even though the values – expressed by personnel and in policy documents – are shared, there are differences in how WT is perceived and justified in relation to these values. In particular, the analysis shows that while the values stressed in the policy documents are not questioned by the interviewed personnel, the justifications of WT concerning these values are *situated* [26, 27].

In the policy documents, the use of WT is justified in relation to the aims of managing the challenges of insufficient economic and human resources in care for older adults. However, the interviews illustrate how the staff draw upon the higher principle of efficiency and optimisation but use it to claim principles related to different worlds of worth. For instance, using WT may involve efficient time use for the publicly funded organisation, but it also provides time for building social relationships.

Social relationships as a higher principle is stressed by the interviewees, but also in the policy documents where the possibility of ‘freeing up time’ for social interaction is advocated from the employee perspective, as human relationships are viewed as a rewarding aspect of the job. However, in the interviews, this is situated by discussing whether or not the use of WT may, in practice, improve or challenge the possibility of working towards this higher principle. On the one hand, WT is justified by reallocating time spent on different tasks so that more time can be spent on social interactions. Unlike how this is talked about in the policy documents, as a means to provide more time for social relationships, some interviewees also see the technology itself as a facilitator of relationship-building. On the other hand, personnel discuss how WT may substitute for human interaction in some situations, and thereby rather detract from the attainment of this value. This corroborates previous research by Frennert [18] and Lydahl [19], who identify concerns raised about the potential loss of core care values with the introduction of digital welfare technologies, such as a threat to the possibility of building relationships. Upholding the value of social relationships, and the evaluation of WT in relation to the value of social relationships, is thereby situated in the everyday practice and experiences of the health and social care workers.

Moreover, it should be noted that justifications are also situated in relation to the specific tasks that belong to different occupational groups. The administrators, much like the producers of policy documents, do not work directly with older adults and tend to focus more on how WT can enhance efficiency and optimisation within the organisation. In contrast, managers − who align more closely with care workers than administrators − justify the use of WT by highlighting its potential to support values such as dignity, freedom, engagement, participation, and social connection. This difference in perspective likely stems from the fact that many managers have previously worked as care workers themselves, maintaining a closer connection to the day-to-day realities of care workers’ tasks.

Finally, it could be noted how justifications for WT – both in policy documents and by the interviewees – refer to the value of good working conditions: using WT to reduce work hazards and stress. The presented justifications suggest that these technologies can handle routine tasks, thereby relieving care staff. In the interviews with personnel, however, some also referred to this value when problematising the use of WT, as it was also associated with technology-related stress and increased work stress. As this example illustrates, the value of good working conditions is shared but the evaluation of the technology differs due to situated judgements.

In summary, this points to the importance of taking the situated judgements of different groups of health and social care workers into account when evaluating the use of WT and discussing which values should be at the core of policies guiding this development. It is argued that attending to professional judgements when introducing welfare technologies is vital for its successful implementation [37].

### Hierarchies of worth

As already demonstrated, care workers and managers more often − compared to the policy documents – justify the use of WT by upholding values affiliated with the domestic world of worth, i.e., building relationships, compassion, caring for others, solidarity, socialising, and shared enjoyment. The findings show how, in care workers’ accounts, the values related to the industrial world of worth are constantly negotiated and situated through the domestic and civic worlds of worth. Efficiency never seems to be an end goal, but rather a means to achieve something else: such as freeing up time to allow care workers to focus on care. This involves a downplaying of industrial values in favour of values associated with the domestic world of worth.

Although the justifications presented in policy documents and interviews reflect shared, rather than conflicting, values, it seems that the relative weights given to different values differ. The analysis thereby shows a significant hierarchisation of value, wherein effectiveness and efficiency are positioned below values associated with the domestic and civic spheres. Taking both the situated judgements and these hierarchies of worth into account, it could be argued that the sharing of values is not as important for the practical introduction of WT as is the negotiation of these values taking place at the organisational level. This is also where concerns regarding important values at risk of being lost are most likely to be raised.

## Conclusions

In justifying the use of WT in Swedish care for older people, the interviewed managers, administrators and care workers upheld values of optimisation and efficiency, activation and participation, dignity and freedom, social relationships, and good working conditions. All of these values are also found in the analysed policy documents and correspond to the higher principles of the industrial, civic, and domestic worlds of worth as described by Boltanski and Thévenot [26]. In conclusion, personnel and policy documents appear to be acting in shared worlds of worth, and show similar judgements on what higher principles should be guiding the organisation of care for older people.

However, the study also demonstrates that even though the values – expressed by personnel and in policy documents – are shared, there are differences in how WT is perceived and justified in relation to these values. While the values stressed in the policy documents are not questioned by the interviewed personnel, the care workers’ justifications are rooted in their occupational experiences, providing examples of situated judgements of the use of WT. Notably, care workers employ efficiency principles to advocate for other values, such as social relationships. Furthermore in comparison to the policy documents, the care workers emphasise values associated with the domestic and civic worlds of worth, downplaying values of optimisation and efficiency. This is described as a hierarchy of worth.

A basic assumption for this study is that the use of WT does not depend on the technologies themselves, but on various contextual factors among which the values upheld by the users of technology are significant. Taking both the situated judgements and the hierarchies of worth into account, it could be argued that – even though it is important that workers share the values promoted in policy documents – it is not as important for the practical introduction of WT as is the negotiation of these values taking place at the organisational level.

Limitations of this study involve potential bias based on the selection of municipalities and interviewees. The sample of interviewed personnel is based on self-selection, where certain groups of interviewees may be lacking from the sample with potential consequences for which values are represented in the analysis. With a larger sample, including a larger number of municipalities with a greater geographical distribution across Sweden, it is possible that more differences could have been discerned in comparisons between municipalities. By studying a larger number of health and social care workers, more differences nel and in policy documents are similar, conflicting values may be more prevalent if one were to look at the governing politicians at the municipal level. Future research may address these methodological issues, and investigate the values represented among politicians and in governing documents at the municipal level.

In future political strategy work, we find it important that politicians and civil servants consider the situated judgements of care workers. This means developing more bottom-up strategies, with the situated judgements and values prioritised by care workers as a starting point. Doing so requires an acknowledgement of the existing value hierarchy, where values of optimisation and efficiency are esteemed, but to accommodate higher principles rather than as end goals.

## Supplementary Information

Supplementary Material 1.

## Data Availability

The datasets generated and/or analysed during the current study are not publicly available due to privacy concerns.

## Abbreviations

SALAR: Swedish Association of Local Authorities and Regions
WT: Welfare technology/technologies
