# Supplementary material for: Values of welfare technologies: a qualitative study of how employees in Swedish care for older adults understand and justify the use of new technology
Source: BMC Health Serv Res. 2024 Dec 6;24:1555. doi: 10.1186/s12913-024-12053-1 (PMC11622466; doi:10.1186/s12913-024-12053-1)
Supplement: Supplementary file 1 — Supplementary Material 1. [file 12913_2024_12053_MOESM1_ESM.docx]

**Combined interview guide**

**Background data:**

Name, position, gender, how long you have been at the workplace/had the position you hold

**Introductory questions**

- How long have you worked in the workplace?

- What are your main tasks?

- What is most important for you to feel that you have done a good job?

- What is good care to you?

- What enables you to provide good care?

- What prevents you from providing good care?

- What role do you think technology has in good care?

**Technology**

- How would you describe welfare technology to someone who has not heard of it before?

- Were you involved in the introduction of welfare technology in your workplace? How did it happen?

- How/ in what way is welfare technology part of your work?

- How is welfare technology related to your other work tasks?

- In which contexts/situations do you encounter welfare technology?

- What do you do with welfare technology?

- Is the support/service you provide different with welfare technology?

- Have you encountered any problems when using welfare technology? What have you done then?

- Does the technology make your work easier? How?

- Does the technology complicate your work? If so, how?

- Do you think your work has changed in any way since you started working with welfare technology?

- What do you feel that users/service users think about welfare technology?

- Does it happen that users/service recipients resist welfare technology? What do you do then?

- Are special conditions needed to be able to work with welfare technology?

- What support and training have you received to be able to work with welfare technology?

For managers/ decision-makers:

- Why did you introduce the described digital technologies?
- Would you say that this/these digitalization initiatives have had impact on how you organize work in your company/business (administration, communication, production/logistic, product development)?
- Could you describe whether it has meant that your staff have to share information, rotate responsibility, divide their tasks differently (Functional flexibility).
- Are there any new work tasks/units in your organization, or are you getting rid of work tasks/units?
- Does it mean that you have to get rid of staff or perhaps hire new staff as well?
- Are you drawing advantage of manpower located outside your organization (crowd sourcing, telecommuting etc.)
- Would you describe these changes as being in accordance with the plan or are there any un-intentional consequences that you have to manage? (e.g. quality concerns, coordination)
- What would you say is your main challenge related to how you organize work so that it draws advantage of the digitalization that you have described?
- Would you say that your use of digital technologies have had any impact on the work content in your company?
- Is this an impact that foster specific demands on competence? Did you already have demanded competences?
- Where do you find the competence/skills you need in order to manage the digitalization that you have described?
- Would you say that there has been a change in where you find the competence you need?
- How would you describe the staff that do the work (e.g. permenant staff, temporary staff, new forms av employments/contracts).
- What is the main reason behind these changes?
- would you say that you are coping or struggling with any specific challenges?

**Values**

- In what contexts do you think welfare technology can be good?

- Can you give an example of a specific situation?

- In which contexts do you think welfare technology can be bad?

- Can you give an example of a specific situation?

- What do you think welfare technology can contribute?

- What do you think can be lost with welfare technology?

- Can you think of any arguments against introducing welfare technology?

- What are the arguments in favour of introducing welfare technology? / Why should welfare technology be introduced?

- How is the discussion in the college about welfare technology?

- Have you experienced any conflicts in relation to welfare technology in the workplace? What have they been about?
